# Prefibrotic myelofibrosis: treatment algorithm 2018

**DOI:** 10.1038/s41408-018-0142-z

**Published:** 2018-11-07

**Authors:** Guido Finazzi, Alessandro M. Vannucchi, Tiziano Barbui

**Affiliations:** 1 Hematology and Bone Marrow Transplant Unit, ASST Papa Giovanni XXIII, Bergamo, Italy; 2 Centro Ricerca e Innovazione delle Malattie Mieloproliferative (CRIMM), Azienda Ospedaliera–Universitaria Careggi, Firenze, Italy; 3 FROM Research Foundation, ASST Papa Giovanni XXIII, Bergamo, Italy

## Abstract

Prefibrotic myelofibrosis (pre-PMF) is a distinct entity among chronic myeloproliferative neoplasm diagnosed according to the revised 2016 WHO classification. The clinical picture is heterogeneous, ranging from isolated thrombocytosis, mimicking essential thrombocythemia (ET), to symptoms of high-risk PMF. Retrospective studies showed that survival of patients with pre-PMF is worse than that of ET and better than overt PMF. Whilst a specific prognostic score is lacking, the International Prognostic Scoring System is able to predict survival in pre-PMF patients, yet failing to separate intermediate-1 and -2 groups, and can be used in clinical practice. Each patient should be evaluated for, and interventions adapted to, both life-expectancy and the risk of bleeding and thrombosis. In low-risk patients with expected long survival, observation only is recommended; in cumulated intermediate-1 and -2 risk cases, whose median survival is projected at more than 10 years, treatment is based on symptoms; in high risk cases, with median survival lower than 5 years, intensive management is required. A pragmatic approach to address the risk of bleeding and thrombosis includes: no treatment or low-dose aspirin in asymptomatic patients; aspirin or oral anticoagulation if previous arterial or venous thrombosis, and hydroxyurea as first-line cytoreduction in case of thrombocytosis or leukocytosis.

## Introduction

Since 1976, a group of European pathologists described a subtype of chronic myeloproliferative neoplasms (MPN) that was characterized by megakaryocytic and granulocytic proliferation, with atypical megakaryocyte morphology, but without any significant bone marrow fibrosis^1,2^. This variant, called prefibrotic myelofibrosis (pre-PMF), was formally introduced in the previous 2001 and 2008 World Health Organization (WHO) classifications of tumors^3,4^, and defined as a distinct clinico-pathologic entity in the recent 2016 revision^5^. Pre-PMF is currently accepted as a separate entity among MPN with unique clinical characteristics and outcomes in respect to both overt PMF and from essential thrombocythemia (ET)^6^. In the present paper, we will review current concepts in diagnosis and clinical aspects of pre-PMF and will provide suggestions for prognostic stratification and treatment decisions making.

## Diagnosis

The 2016 WHO classifications of tumors defined the current criteria for the diagnosis of pre-PMF^5^. On histopathology grounds, it is characterized by increased cellularity in relation to age due to granulopoietic and megakaryocytic proliferation, along with precursors’ excess of the former and atypia of the latter, with peculiar bulbous-shaped nuclei and frequent formation of clusters. It may be accompanied by a mild increase in reticulin fibers defined as grade 0 or 1 on a scale of 0 to 3 according to the European Consensus^7^. Conversely, fibrosis of grade 2 or 3 defines overt MF.

The lack of fibrosis in the early phases as well as a clinical onset sometimes characterized by isolated thrombocytosis can lead to pre-PMF being misdiagnosed as ET. Given that bone marrow histology shows different morphological pictures among the two different categories of MPN, its role is crucial in diagnosing pre-PMF. In particular, ET differs from pre-PMF in that cellularity is usually preserved, granulopoiesis and erythropoiesis are in regular ratio and not expanded, and megakaryocytes, although significantly increased, show no or only mild atypia, with mature hyperlobulated nuclei^2,5^.

Finally, diagnosis of pre-PMF should include at least one of the following minor criteria: (a) anemia not attributed to a comorbid condition; (b) leukocytosis ≥11 × 10^9^/L; (c) palpable splenomegaly; (d) serum lactate dehydrogenase (LDH) level increased to above upper normal limit of institutional reference range^5^.

## Clinical phenotype and outcome

Several studies demonstrated that the clinical picture and prognosis of patients with pre-PMF are different from those with ET or overt PMF^8–16^. These studies compared patients with pre-PMF either with those with ET^8,13^ or with overt PMF^14–16^, and deserve to be described separately.

### Studies comparing pre-PMF with ET

In a series of 1104 patients who were previously classified as ET, the diagnosis was reviewed following a strict application of the 2008 WHO classification^8^. The diagnosis of ET was confirmed in 891 patients (81%) and was revised to pre-PMF in 180 (16%) cases. A comparative analysis of presenting clinical and laboratory data revealed significant higher leukocyte and platelet count, lower hemoglobin level, higher LDH level and circulating CD34^+^ cell count, and more frequent palpable splenomegaly in patients with pre-PMF than in those with ET. Patients with pre-PMF exhibit a more frequent evolution to overt myelofibrosis and acute leukemia and inferior overall survival in comparison with ET, as well as with normal population. The respective death, leukemia, and overt myelofibrosis incidence rates per 100 patient-years for pre-PMF compared with ET patients were 2.7 and 1.3% (relative risk [RR], 2.1; *P* < 0.001), 0.6 and 0.1% (RR, 5.2; *P* = 0.001), and 1 and 0.5% (RR, 2.0; *P* = 0.04).

These findings have been confirmed in other studies^9–11,13^ and in a recent analysis of 269 patients with ET and 109 with pre-PMF, reclassified according to the 2016 WHO criteria^12^. Similar differences of the clinical phenotype at presentation between the two groups were reported. *CALR* mutations were more frequent in pre-PMF than in ET (35.8% vs 17.8%, *P* < 0.001). Pre-PMF patients had shorter overall survival (*P* < 0.001) and a trend to a higher incidence of leukemic evolution (*P* = 0.067) compared to ET patients.

### Studies comparing pre-PMF with overt PMF

A multicenter study evaluated the clinical and hematologic presentation, the molecular profile, and the outcome of a cohort of patients with a previous diagnosis of PMF^15^. After revision based on the 2016 WHO criteria, 278 patients were classified as pre-PMF and 383 patients as overt PMF. Compared with pre-PMF, overt PMF was enriched in patients with anemia, thrombocytopenia, leukopenia, higher blast count, constitutional symptoms, large splenomegaly, and unfavorable karyotype. The types of driver mutations were similarly distributed between the two categories, whereas selected mutations comprising the high mutation risk (HMR) category (any mutations in ASXL1, SRSF2, IDH1/2, EZH2) were more represented in overt PMF. More patients with overt PMF were in higher International Prognostic Scoring System (IPSS)^17^ risk categories at diagnosis, and the frequency increased during follow-up, suggesting greater propensity to disease progression compared with pre-PMF. Median survival was significantly shortened in patients with overt PMF than in those with pre-PMF (7.2 vs 17.6 years). Compared with a reference population of patients with ET, survival was progressively shortened depending on fibrosis grade, with hazard ratio (HR) of 1.8, 2.8, 5.3, and 6.2 for fibrosis grade 0, 1, 2, and 3, respectively.

The different clinical presentation and outcome of the two entities was confirmed by a single center study that included 63 patients with pre-PMF and 404 with overt PMF^16^. Of interest, in the particular study, the survival difference between pre-PMF and overt PMF was most evident in high/intermediate-2 DIPSS-plus risk groups^18^. Pre-PMF and overt PMF were similar in their spectrum of non-driver HMR mutations and no difference in survival was observed when patients with grade “0” were compared to those with grade 1 reticulin fibrosis (*P* = 0.96) and when those with grade 2 were compared with grade 3 (*P* = 0.08).

In another study including 132 patients with pre-PMF and 551 with overt PMF the median survival was not reached in pre-PMF, while it was 16.6 years in overt PMF patients (<0.001). 98%, 81%, and 56% of patients with pre-MF, overt PMF with early and overt PMF with advanced bone marrow fibrosis, respectively, were alive at 10 years from the diagnosis (*P* < 0.001)^14^.

## Risk factors and risk stratification

**Fig. 1:**
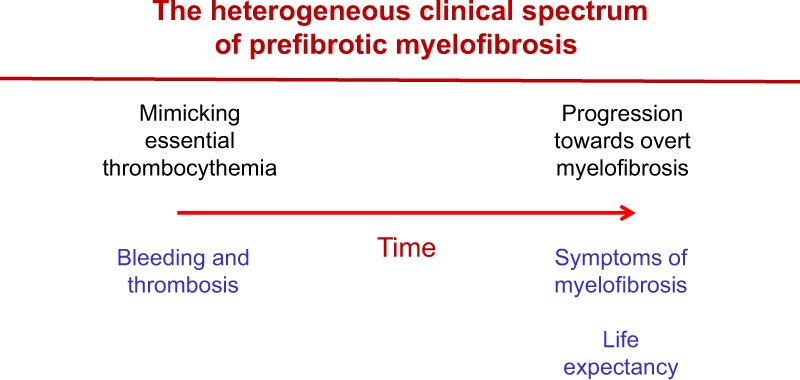
The heterogeneous clinical spectrum of prefibrotic myelofibrosis

The clinical picture of pre-PMF patients is heterogeneous, ranging from isolated thrombocytosis, mimicking ET^8^, to high-risk PMF^15^. The disease is progressive and tends to move over time to more severe forms of PMF (Fig. 1)^8,14^. However, the rate of progression is variable and not all patients with pre-PMF will necessarily develop an overt disease^8,14,15^. Patients with pre-PMF should be evaluated both for their life expectancy and the risk of major thrombotic and bleeding complications.

### Life expectancy

In the multicenter study reported above^15^, survival in patients with pre-PMF was predicted by the IPSS score, but the curves of intermediate-1 and intermediate-2 patients did not differ significantly (*P* = 0.205). Using the low risk as the reference category, the HR for intermediate-1, intermediate-2, and the high risk category was 5.3 (95% CI, 2.4–11.8), 12.2 (95% CI, 5.0–30.1), and 34.8 (95% CI, 15.7–77.2), respectively.

The impact of driver and non-driver mutations on OS was also evaluated^15^. In pre-PMF, CALR type 1 mutation was the most favorable, with median survival of 27.7 years. Using this as the reference category, the HR for reduced survival was 3.8 (95% CI, 1.3–10.6; *P* = 0.013) for merged JAK2V617F/MPLW515 mutations, 6.1 (95% CI, 1.6–23.3; *P* = 0.008) for CALR type 2, and 22.8 (95% CI, 7.1–73.3; *P* < 0.0001) for triple negative patients. A HMR and low mutation risk (LMR) status was defined, respectively, by the presence of at least 1 mutated gene or the absence of any mutation. The HMR category was associated with significantly shorter survival in pre-PMF. Median (range) survival was 8.3 years (3.6–13.0 years), compared with 20.2 years (13.1–27.3 years) for the LMR category (*P* < 0.0001).

### Risk of bleeding and thrombosis

Two studies showed that patients with pre-PMF present a risk of vascular events similar to that of ET. In the first paper, the rates of major thrombosis in pre-PMF and ET patients were 1.9% and 1.7% patients per year, respectively^8^. In the second study, the 10-year cumulative incidence of thrombosis was 18.5% in pre-PMF and 18% in ET^12^. There are only a few studies that evaluated specifically risk factors of thrombosis in pre-PMF patients^19^. In a study of 264 patients, leukocytosis at diagnosis was a significant risk factor for overall (*P* = 0.005, HR 1.15) and arterial thrombosis (*P* = 0.047, HR 1.12)^20^. Another study focused on the impact of leukocytosis on thrombotic events over time. Among 189 ET and pre-PMF patients, elevated baseline leukocyte count at diagnosis was a significant risk factor for thrombosis, but leukocyte count during follow-up had no impact. This result mainly applied to pre-PMF since the majority of ET patients had normal leukocyte count. The platelet counts and hemoglobin level were not significant risk factors of thrombosis both at diagnosis and during follow-up^21^.

At variance of thrombosis, one study demonstrated that major bleeding during follow-up occurred more frequently in pre-PMF than in ET patients, at a rate of 1.39% and 0.79% patients per year, respectively (*P* = 0.039)^22^. In multivariable analysis, predictors of bleeding included diagnosis of pre-PMF, leukocytosis, previous hemorrhage, and aspirin therapy. The particular study revealed that major bleeding associated with thrombocytosis might be relatively specific to pre-PMF, potentially exacerbated by low-dose aspirin use^22^. The reticulin grade was found to be an independent risk factor of bleeding during follow-up in a prospective study of ET patients diagnosed according to the Polycythemia Vera Study Group (PVSG) criteria^11,23^, raising the question of whether those patients had thrombocythemic manifestations of pre-PMF. In another study, however, the 10-year cumulative incidence of bleeding was 14.9% in pre-PMF and 19.6% in ET patients, without significant difference between the two groups^12^.

## Treatment algorithms

A comprehensive management of pre-PMF patients should be addressed both to improving life-expectancy and to prevent vascular complications.

### Improving survival

**Fig. 2:**
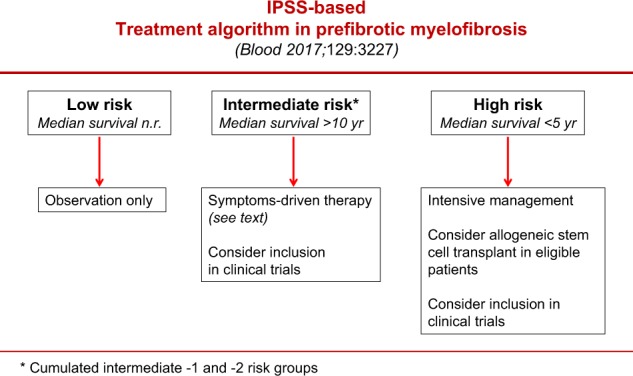
Treatment algorithm in prefibrotic myelofibrosis according to IPSS. n.r. not reached

Several prognostic models for survival have been defined for overt PMF^17,18,24–28^, but none of them have been validated also for pre-PMF. Only the IPSS has been separately analyzed in 278 patients with pre-PMF, showing that the score predicted survival, yet it failed to accurately separate intermediate-1 and intermediate-2 groups of patients^15^. With this limitation, and waiting for better prognostic models, IPSS remains a useful tool to separate pre-PMF patients at least in low, intermediate (cumulating -1 and -2), and high risk categories with different median survival and therapeutic needs (Fig. 2).

In pre-PMF asymptomatic patients with low-risk disease, there is no evidence to support the value of any therapeutic intervention and observation alone is usually recommended^29,30^. Patients at intermediate-risk might require palliative therapy for anemia, splenomegaly, or constitutional symptoms^29,30^. If cytoreductive treatment for the reduction of leukocytosis or thrombocytosis is indicated, the first-line drug of choice is hydroxyurea^29^. Busulfan can be used in elderly individuals who are intolerant to hydroxyurea. Ruxolitinib can be considered in pre-PMF patients with intermediate or high-risk disease when splenomegaly or systemic symptoms in need of treatment are present^31,32^. In a recent study, long-term ruxolitinib therapy reversed or delayed bone marrow fibrosis progression in advanced MF^33^, but whether these effects apply also to pre-PMF patients is undetermined. In pre-PMF patients with intermediate-2 disease, median survival was superimposable to intermediate-1 and projected at >10 years and, thus, allogenic stem cell transplantation (ASCT) might be inappropriate^15,34^. At variance, pre-PMF patients classified at high risk according to IPSS have a dismal median survival, lower than 5 years and similar to that of high-risk overt PMF^15^. These patients, that account for 12% of the entire pre-PMF cohort^15^, require intensive management, including consideration of ASCT in eligible cases^34^.

### Preventing major bleeding and thrombosis

**Fig. 3:**
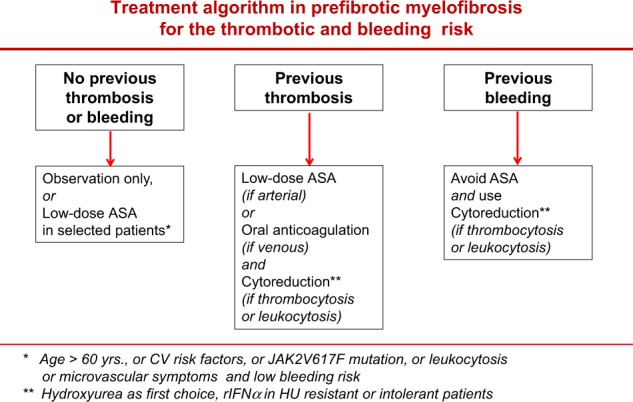
Treatment algorithm in prefibrotic myelofibrosis according to the risk of thrombosis and bleeding. ASA aspirin, CV cardiovascular, rIFNα recombinant interferon alpha

Specific prognostic scores for predicting the risk of bleeding and thrombosis in PMF patients are not available. The IPSET-T^35^, that is the prognostic system for thrombosis currently recommended for ET^29^, specifically excluded from the analysis patients re-classified as pre-PMF according to the WHO criteria. Thus, for the time being, a pragmatic approach can be suggested (Fig. 3). In pre-PMF patients without a previous history of bleeding or thrombosis, no treatment is the first option. In selected patients with clinical or laboratory features that are very likely to point to a high risk of thrombosis in pre-PMF^20,21^, or in other MPNs^19,29,36^, such as age >60 years, general cardiovascular risk factors (hypertension, diabetes, smoking), leukocytosis and presence of JAK2V617F mutation, or with microvascular symptoms (e.g., erythromelalgia), low-dose aspirin (100 mg daily) can be considered, provided that a high bleeding risk is ruled out. In patients with previous vascular events, low-dose ASA in arterial or oral anticoagulation in venous thrombosis are recommended. Cytoreductive therapy should be added in the presence of thrombocytosis or leukocytosis and hydroxyurea is the drug of choice. In patients with previous bleeding, or high bleeding risk, such as extreme thrombocytosis with acquired von Willebrand disease^37–39^, ASA should be avoided and HU is the recommended treatment. In pre-PMF patients requiring cytoreduction but resistant or intolerant to HU^40^, recombinant interferon-alpha is indicated^29,41,42^. Anagrelide is not recommended^30^, since this drug was associated with the development of post-ET myelofibrosis in the randomized PT-1 clinical trial^43^, which based the diagnosis of ET on PVSG criteria and thus did not exclude pre-PMF patients^11^. On the other hand, in a strictly WHO-defined cohort of ET patients a prospective randomized noninferiority phase 3 study provided no evidence for transformation into myelofibrosis^44^.

## Future advances

It is likely that, in the next future, we will significantly change the way we predict prognosis and individualize treatment for pre-PMF patients^45^. We need prospective studies of well-defined cohorts of patients with initial, treatment-naive bone marrow biopsies and centralized evaluations to overcome the limits of the current knowledge and to develop an unified prognostic score specifically designed for this disease. Prognostication in the field of PMF (either prefibrotic or overt) is actively changing, moving towards more comprehensive and integrated scores, as the MIPSS70/MIPSS70-plus^26,27^, that includes the high molecular risk profile whose negative influence is maintained in the setting of pre-PMF, and the GIPSS^28^, which is entirely genetic-based. We reasonably expect that these advances can help us in shaping our choices in terms of management and treatment of this relatively “new-born” entity in the spectrum of MPN.
